# Association between Proton Pump Inhibitor Therapy and *Clostridium difficile* Infection: A Contemporary Systematic Review and Meta-Analysis

**DOI:** 10.1371/journal.pone.0050836

**Published:** 2012-12-07

**Authors:** Imad M. Tleyjeh, Aref A. Bin Abdulhak, Muhammad Riaz, Faisal A. Alasmari, Musa A. Garbati, Mushabab AlGhamdi, Abdur Rahman Khan, Mohamad Al Tannir, Patricia J. Erwin, Talal Ibrahim, Abed AlLehibi, Larry M. Baddour, Alex J. Sutton

**Affiliations:** 1 Department of Medicine, King Fahad Medical City, Riyadh, Saudi Arabia; 2 College of Medicine, AlFaisal University, Riyadh, Saudi Arabia; 3 Division of Infectious Diseases, Mayo Clinic, Rochester, Minnesota, United States of America; 4 Division of Epidemiology, Mayo Clinic, Rochester, Minnesota, United States of America; 5 Department of Internal Medicine, University of Missouri-Kansas City, Kansas City, Missouri, United States of America; 6 Research and Scientific Publication Center, King Fahad Medical City, Riyadh, Saudi Arabia; 7 Department of Internal Medicine, University of Toledo Medical Center, Toledo, Ohio, United States of America; 8 Mayo Medical Library, Mayo Clinic, Rochester, Minnesota, United States of America; 9 Department of Orthopedic and Trauma Surgery, Hamad Medical Corporation Doha, Qatar; 10 Department of Health Sciences, University of Leicester, University of Leicester, Leicester, England; Charité, Campus Benjamin Franklin, Germany

## Abstract

**Introduction:**

Emerging epidemiological evidence suggests that proton pump inhibitor (PPI) acid-suppression therapy is associated with an increased risk of *Clostridium difficile* infection (CDI).

**Methods:**

Ovid MEDLINE, EMBASE, ISI Web of Science, and Scopus were searched from 1990 to January 2012 for analytical studies that reported an adjusted effect estimate of the association between PPI use and CDI. We performed random-effect meta-analyses. We used the GRADE framework to interpret the findings.

**Results:**

We identified 47 eligible citations (37 case-control and 14 cohort studies) with corresponding 51 effect estimates. The pooled OR was 1.65, 95% CI (1.47, 1.85), I^2^ = 89.9%, with evidence of publication bias suggested by a contour funnel plot. A novel regression based method was used to adjust for publication bias and resulted in an adjusted pooled OR of 1.51 (95% CI, 1.26–1.83). In a speculative analysis that assumes that this association is based on causality, and based on published baseline CDI incidence, the risk of CDI would be very low in the general population taking PPIs with an estimated NNH of 3925 at 1 year.

**Conclusions:**

In this rigorously conducted systemic review and meta-analysis, we found very low quality evidence (GRADE class) for an association between PPI use and CDI that does not support a cause-effect relationship.

## Introduction

Proton pump inhibitors (PPIs) are one of the most prescribed groups of drugs globally [1]. PPIs are effective for the treatment of all acid-related disorders. They are also indicated ICU patients with coagulopathy, patients on mechanical ventilation, and patients with history of peptic ulcer disease, (particularly those on NSAID or antiplatelet therapy) [2].

The use of PPIs has increased dramatically [1] despite concerns that PPIs are overprescribed both in primary care [3] and in hospitals, both in the in-patient setting [4]–[7] and on discharge [8]. Moreover, concerns have been raised about the potential long-term effects of these drugs. PPIs have been associated with significant interaction with other drugs [9], [10] and fractures [11], interstitial nephritis [12], pneumonia [13] and enteric infections [14], [15], namely *Clostridium difficile* infection (CDI).

CDI has recently emerged as a major public health problem with current estimates suggesting a point prevalence of 13.1/1000 in-patient population [16]. Studies have reported increases in both incidence and mortality of CDI [17]–[20]. The increase in incidence of CDI has been attributed to an aging population, increase in use of antibiotics and acid suppressive drugs. PPIs are postulated to increase the proliferation of spores and change the acidic milieu of the stomach that permits spores to survive intraluminally.

The role of gastric acid suppression therapy has gained much interest recently as a risk factor for CDI. Four recently published meta-analyses have suggested an association between gastric acid suppression therapy with proton pump inhibitors (PPI) and CDI [15], [21], [22], [23]. The United States Food and Drug Administration (FDA) recently warned the public about a possible association between CDI and PPI use [19]. Nevertheless, these reviews had important limitations such as missing a large number of published studies [15], [19], [22], [23], using only unadjusted data from observational studies [15], [22], [23], not exploring heterogeneity and the effect of publication bias and over-interpreting the findings. We, therefore, performed a systematic review and meta-analysis that addressed the role of PPIs in CDI. We used the MOOSE [24] and PRISMA guidelines [25] for reporting systematic reviews. We include new studies published after the previous meta-analyses and added unique approaches to adjust for publication bias as well as explore the potential effect of unknown confounders. We use the Grades of Recommendation, Assessment, Development and Evaluation (GRADE) framework [26] to interpret our findings.

## Methods

### Study Search Strategy

The search strategy and subsequent literature searches were performed by a medical reference librarian (PJE) with 38 years of experience. The initial strategy was developed in Ovid MEDLINE (1990 through January 2012), using MeSH (Medical Subject Headings) controlled vocabulary, and then modified for Ovid EMBASE (1990 through January 2012). The search was intended to capture all acid suppression studies. Primary terms were: enterocolitis, pseudomembranous/AND the therapeutic agents of interest: explode omeprazole, explode proton pump inhibitors, anti-ulcer agents, and explode histamine H_2_ antagonists (Explode allows including all of the specific drugs, without having to use all of the various terms, synonyms, brands and generic names.) Articles were limited to randomized controlled trials, cohort studies, and/or case-control studies. The same process was used with Ovid EMBASE with alterations as necessary to accommodate EMBASE’s more granular subject headings. ISI Web of Science and Elsevier Scopus use textwords: (difficile OR pseudomembranous OR pseudo-membranous) AND (omeprazole OR “proton pump” OR ranitidine OR h2 OR h-2 OR “acid suppression” OR antacid*)) AND (random* OR trial* OR blind* OR cohort* OR controlled OR prospective).

There was no restriction on language. All results were downloaded into EndNote 7.0 (Thompson ISI Research soft, Philadelphia, Pennsylvania), a bibliographic database manager, and duplicate citations were identified and removed. Two authors (A.B.A. and F.A.) independently assessed the eligibility of identified studies.

### Study Selection

To be included, a study had to: (1) be an analytical study; and (2) have examined the association between PPI use and incidence of CDI.

### Data Collection

A data collection form was developed and used to retrieve information on relevant features and results of pertinent studies. Two reviewers (A.B.A. and F.A.) independently extracted and recorded data on a predefined checklist. Disagreements among reviewers were discussed with two other reviewers (I.M.T. and M.A.), and agreement was reached by consensus. Data included the following: study characteristics (i.e., country and year of study), characteristics of the study, PPI intake definition and ascertainment, and outcome. We also collected adjusted effect estimates and 95% confidence intervals (CI) based on the multivariable regression model used in each study, and the list of variables considered for inclusion in the multivariate analysis.

We used the Newcastle-Ottawa Quality Assessment Scale for cohort and case-control studies [27] which is intended to rate selection bias, comparability of the exposed and unexposed groups of each cohort, outcome assessment, and attrition bias. Two reviewers (A.B.A. and F.A.) independently assessed the methodological quality of selected studies using the Newcastle-Ottawa Quality Assessment Scale for cohort and case-control studies. Disagreement among reviewers was discussed with 2 other reviewers (I.M.T. and M.A.), and agreement was reached by consensus.

We used the GRADE framework to interpret our findings. The Cochrane Collaboration has adopted the principles of the GRADE system [28] for evaluating the quality of evidence for outcomes reported in systematic reviews.

For purposes of systematic reviews, the GRADE approach defines the quality of a body of evidence as the extent to which one can be confident that an estimate of effect or association is close to the quantity of specific interest. Quality of a body of evidence involves consideration of within-study risk of bias (methodological quality), directness of evidence, heterogeneity, precision of effect estimates and risk of publication bias.

### Statistical Analyses

#### Meta-analyses

The primary effect measures used in the meta-analysis were Odds Ratios (OR) (46 observations), and Hazard Ratios (HR) (5 observations) which were assumed to reasonably estimate the same association between CDI and PPIs because of low CDI incidence and are pooled together. Adjusted effect estimates were primarily used for this analysis. Unadjusted effect estimates were used as alternatives if studies did not observe an association on univariate comparison and did not therefore pursue adjustment or did not report adjusted estimates. We performed meta-analyses for all studies together and separately for different subgroups such as case-control studies and cohort studies.

Effect estimates from all included studies were pooled in a meta-analysis using the DerSimonian and Laird random effects model [29].

#### Exploring heterogeneity

Homogeneity among studies was estimated by calculation of the variation across studies attributable to heterogeneity rather than chance (I^2^). The influence of a range of a-priori selected study-level and aggregated individual-level parameters on the observed effect estimate was investigated by means of meta-regressions. In these analyses, the log odds ratio from each study was regressed on the potential confounders in univariate and multivariate weighted linear regressions, weighted according to the inverse standard error and the residual between-study variance. Nine potential confounders were considered. Six variables were categorical: design of the study (case-control vs. cohort), country of publication, setting (single center vs. multicenter), method of ascertainment of antibiotic use, method of effect measure (OR vs. RR/HR) and effect estimate (adjusted vs. unadjusted). Three continuous variables were: the impact factor of the journal where the study was published, number of variables the effect measure was adjusted for and proportion of cases that were exposed to antibiotics.

#### Publication bias

The possible influence of publication bias was graphically assessed with the novel method of contour-enhanced funnel plot [30] where log-transformed odds ratios were plotted against standard errors. This method examines whether any funnel plot asymmetry is likely to be due to publication bias compared with other underlying causes of funnel plot asymmetry. The contours help to indicate whether areas of the plot, where studies are perceived to be missing, are where studies would have statistically significant effect sizes or not and thus decrease or increase the evidence that the asymmetry is due to publication bias. The presence of funnel plot asymmetry was also assessed using Egger’s test [31]. To adjust for the impact of publication bias on the pooled effect estimate, we used a novel regression based adjustment method recently suggested by Moreno et al [32]. An adjusted pooled effect estimate for an ideal study of a very large size (i.e. with zero standard error) is obtained from the fitted weighted linear regression equation, plotted with a regression line on the contour enhanced funnel plot. This method of regression is a modified version of conventional Egger’s regression test for publication bias where the log of effect estimate is regressed by its variance rather than the standard error and weights are assigned according to the inverse of the variance. This model has been shown to consistently outperform the conventional trim and fill method [32].

#### Residual confounding

Finally, the possible influence of unknown confounders (residual confounding) was investigated with a rule-out approach described by Schneeweiss [33]. This approach stipulates the influence of a hypothetical confounder and determines what characteristics this confounder must have to fully account for the observed association between use of PPIs and occurrence of CDI. The hypothetical confounder is characterized by its association to PPIs use (OR_EC_, odds ratio of exposure to the confounder) and its association to the outcome (RR_CO_, relative risk of outcome in individuals exposed to the confounder vs. non-exposed). For this analysis, the absolute risk in the pooled non-exposed group was used for conversion of odds ratio to relative risk using the method described by Zhang and Yu [34]. Separate analyses were performed to demonstrate what levels of OR_EC_ and RR_CO_ would be required to fully explain the observed association between PPIs and CDI for different hypothetical prevalence of the unknown confounder (i.e. P_C_ = 0.2, P_C_ = 0.5) before and after adjustment for publication bias as described above.

In all analyses, results associated with p-values <0.05 (two-sided test) were considered statistically significant. All statistical analyses were performed using Stata version12 statistical software (Stata Corp, College Station, Texas).

## Results

### Yield of Search Strategy and Eligible Studies

**Figure 1:**
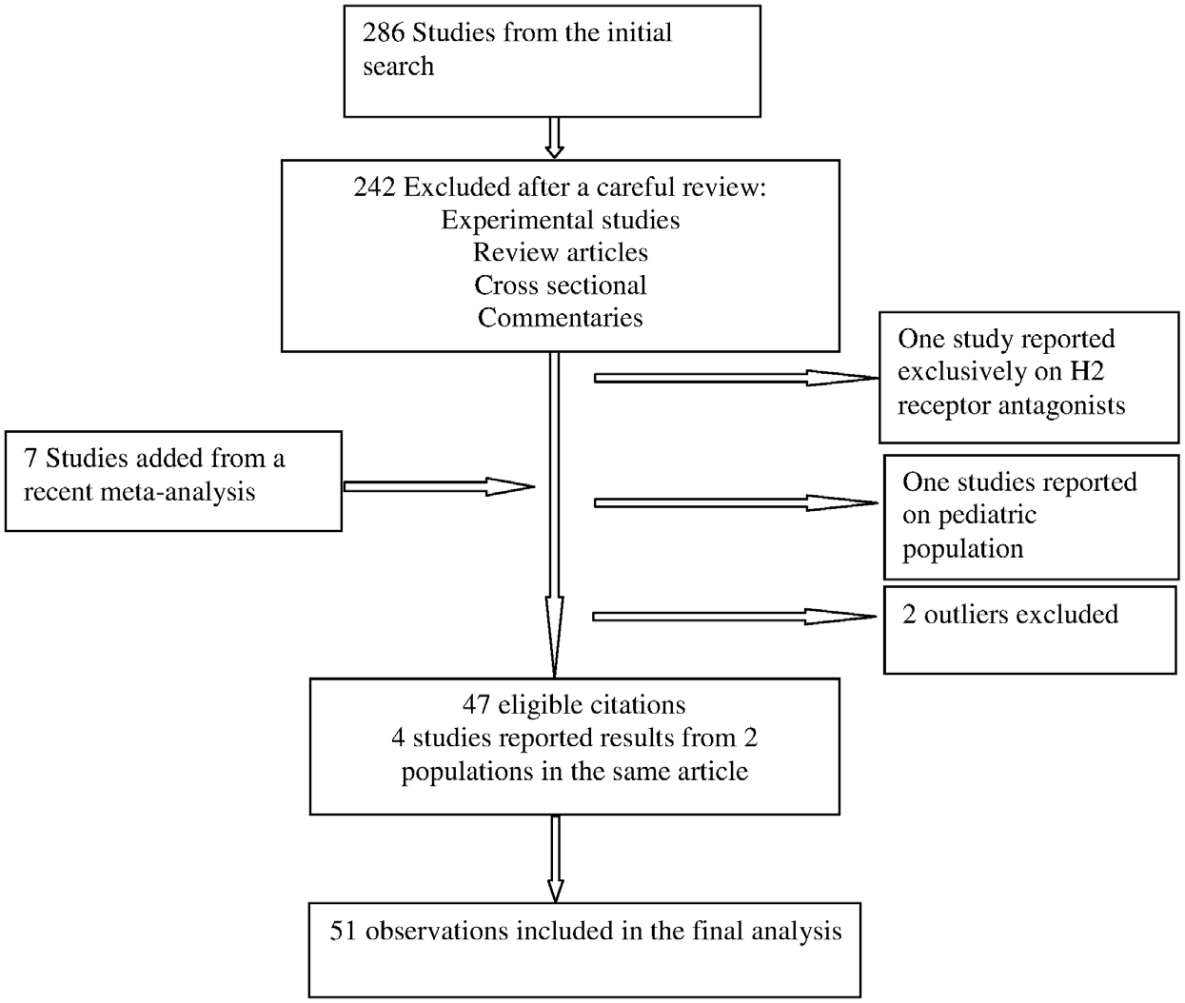
Flow diagram of eligible studies.

The search strategy yielded 287 publications of which 242 were not eligible for inclusion based on title/abstract review. Reference lists of all eligible articles were systematically searched and 7 additional studies were identified that were not captured by our search strategy. A total of 47 citations, 4 of which reported data on 4 different populations, that examined the association between PPI therapy and CDI were eligible for this review. Figure 1 summarizes the study selection process and is presented in the appendix.

### Characteristics of the Included Studies

Table 1 summarizes characteristics of the included studies. Studies were conducted in Asia, Europe, North America (Canada and USA). One citation reported both a case-control and cohort designs on 2 different patient populations [35]. Three citations [36]–[38] reported two different case control analyses per each citation. Forty analyses were single-centre, nine were multi-centre, and two from a general practice research database (GPRD). Thirty seven analyses were of case-control design and 14 were of cohort design. Among these, 8 exclusively addressed community-acquired CDI, 37 hospital-acquired and 6 both hospital- and community-acquired CDI. Table S1 and S2 summarizes the CDI case ascertainment and control or non-exposed group selection method for all studies.

**Table 1:** Characteristics of the Included Studies.

Source	Country	Centers	Setting	Condition	Study Design	Inclusion Criteria	Acid Suppression Therapy
Kutty et al (VA),^1^ 2010	US	Multicenter	Community	Gen Pop	Case-control	Age: ≥18 yr; Community onset CDAD	PPI exposure 3mo prior to test
Kutty et al (D),^1^ 2010	US	Multicenter	Community	Gen Pop	Case-control	Age: ≥18 yr; Durham residence; Community onset CDAD	PPI exposure 3mo prior to test
Southern et al,^2^ 2010	US	Multicenter	Hospital	Abd-surgery pts	Case-Control	Positive CD within 30 d after surgery; diarrhea	PPI: exposure within 10d before surgery
Nath et al,^3^ 1994	CA	Single	Hospital	Hem-oncology pts	Case-control	Adult; In-patient > 3d	Acid suppressive therapy
Jayatilaka et al,^4^ 2007	US	Single	Hospital	Gen In-patient	Case-control	Age >18	PPI pre or during admission
Jayatilaka et al,^4^ 2007	US	Single	Hospital	Gen In-patient	Case-control	Age >18	PPI: post admission
Branch et al,^5^ 2007	US	Single	Hospital	Gen In-patient	Case-control	Age ≥18yr ; In-patient	PPI: 3d before testing or 7d within the last month; before hospitalization
Al-Tureihi et al,^6^ 2005	US	Single	Hospital	LTCF pts	Case-control	Positive CD toxin; documented diagnosis/ ABX for CDAD	PPI
Shah et al (D),^8^ 2000	UK	Single	Hospital	Gen In-patient	Case-control	Age > 65 yr; Gen medical/ elderly care wards	PPI: up to 16wk before diarrhea
Dial et al,^11^ 2005	UK	GPRD	Community	Gen Pop	Case-control	Age ≥18 yr ; At least 2 yrs of records in the GPRD prior to index date; first occurrence of CDAD	PPI: 90d prior to the index date
Akhtar et al,^12^ 2007	US	Single	Hospital	Gen In-patient	Case-control	Afro-American/ Hispanic pts admitted with diarrhea positive CD	PPI: 3m before diarrhea
Dial et al,^13^ 2006	UK	GPRD	Community	Gen Pop	Case-control	All pts with first prescription oral Vancomycin; No previous admission 1yr before index date	PPI: 90d prior to the index date
Asseri et al,^14^ 2008	US	Single	Hospital	Gen In-patient	Case-control	Age ≥18 Yr; Inpt for ≥3 d	PPI: at least 3d before CDAD
Cunningham et al,^15^ 2003	UK	Single	Hospital	Gen In-patient	Case-control	NR	PPI: preceding 2mo
Dubberke et al,^16^ 2007	US	Single	Hospital	Gen In-patient	Case Control	Pts admitted for > 48 hr between study period were included	PPI
Dial et al,^17^ 2004	CA	Single	Hospital	Gen In-patient	Case-control, (P)	In- pts with CDAD; Positive toxin result during or within m after the index admission	PPI : at least 3d before diarrhea ; 3d in hospital if no diarrhea
Loo et al,^18^ 2005	UK	Single	Hospital	Gen In-patient	Case-control	Hospital Acquired CDAD;	PPI: 6wk before diagnosis
Sundram et al,^19^ 2009	UK	Single	Hospital	Gen In-patient	Case-control	Adult Hospital Acquired CDAD	PPI: 6wk prior to onset of CDAD
Howell et al,^29^ 2007	US	Single	Hospital	Gen In-patient	Cohort	Age ≥ 18 yr; LOS ≥3 d; Only first diagnosis	Daily PPI
Dalton et al,^30^ 2009	CA	Multicenter	Hospital	Med/Surgical Subspecialty	Cohort , (R)	Age: ≥18 yr; Minimum 7-d LOS; ABX exposure	PPI Days: 18.7±21.5
Dubberk et al,^31^ 2007	US	Single	Hospital	Gen In-patient	Cohort, (R)	All pts admitted to BJH for more than 48 hours	PPI
Pépin et al,^32^ 2005	CA	Single	Hospital	Gen In-patient	Cohort, (R)	Adult In-patient	PPI
Beaulieu et al,^33^ 2007	CA	Single	Hospital	Medical ICU	Cohort	LOS in ICU>24hr ; Diarrhea >24 hr and positive CD toxin between 2d to 2mo after discharge	PPI
Peled et al,^34^ 2007	IL	Single	Hospital	Gen In-patient	Cohort , (P)	CD testing during 4m period; ABX within 40d prior to diarrhea	PPI
Dial et al,^17^ 2004	CA	Single	Hospital	Med/CT surgical wards	Cohort	Pharmacy database; ABX during study period; positive toxin in the infection control registry	PPI
Linsky et al,^28^ 2010	US	Multicenter	Community & Hospital	Gen Pop	Cohort , (R)	First positive CD toxin, VA health care system use for 1 yr before or after the index CDAD	PPI: Concurrent with CDAD Treatment
Baxter et al,^7^ 2008	US	Single	Hospital	Gen In-patient	Case control	In-patient	PPI: 60d before index date
Debast et al,^20^ 2009	NL	Single	Hospital*	Gen In-patient	Case control	Age:≥18 yr; CDAD	PPI: In the past 3mo
Yearsley et al,^10^ 2006	UK	Single	Hospital	Gen In-patient	Case control, (P)	Age > 18 yr	PPI: 3m prior to study entry
Lowe et al,^9^ 2006	CA	Single	Community	Gen Pop	Case control, (R)	1 hospital admission for CDAD; Age ≥ 66yr; CDAD diagnosis within 60d of ABX therapy	PPI : upto 365d prior to the index date
Novell et al,^21^ 2010		Single	Hospital	Gen In-patient	Case control, (R)	Age:≥18 yr; CDAD	PPI
Hensgens et al,^22^ 2011	NL	Single	Hospital	Gen In-patient	Case control, (P)	All hospitalized patients with CDAD	PPI
Netland et al,^35^ 2011	US	Single	Community & Hospital	Gen Pop	Cohort , (R)	Recurrent CDAD	PPI
Ingle et al,^36^ 2011	Ind-ia	Single	Community & Hospital	Gen Pop	Cohort , (R)	All patients with CDAD	PPI
♦Monge et al,^27^ 2011	Sp-ain	Single	Hospital	NA	Case control	NA	PPI
Jenkins et al,^23^ 2010	UK	Single	Hospital		Case control, (R)	Post Hip/Knee replacement, CDAD	PPI
Shaughnessy et al,^37^ 2011	US	Single	Hospital	Medical ICU	Cohort, (R)	All patients with CDAD diagnosis	PPI
Kim et al,^38^ 2010	CA	Single	Hospital	Gen In-patient	Cohort , (R)	Age:≥18 yr; Recurrent CDAD	PPI
Loo et al,^39^ 2011	CA	Multicenter	Hospital	Gen In-patient	Cohort	Age:≥18 yr; healthcare associated CDAD	PPI
Manges et al,^24^ 2010	CA	Single	Hospital	Gen In-patient	Case control	Nosocomial CDAD	PPI
Kuntz et al,^26^ 2011	US	Single	Community	Gen Pop	Case control, (R)	Community acquired CDAD	Acid suppressive therapy
Naggie et al,^25^ 2011	US	Multicenter	Community	Gen Pop	Case control, (R)	Age:≥18 yr	Acid suppressive therapy
Stevens et al,^40^ 2011	US	Single	Hospital	Gen In-patient	Cohort	Age:≥18 yr; hospital acquired; non-psychiatric ward; exposed to antibiotics	PPI
Dial et al,^46^ 2008	CA	Multicenter	Community	Elderly patients	Case control	Age ≥65, Community Associated CDAD	PPI
McFarland et al,^42^ 2007	US	Multicenter	Both	Gen Pop	Case control	CDAD Diagnosis	PPI
Kazakova et al,^47^ 2012	US	Single	Both	Gen Pop	Case control	CDAD Diagnosis, onset during the pre-outbreak or outbreak periods, hospitalization	PPI
Modena et al,^43^ 2005	US	Single	Both	Gen Pop	Case control	Received at least 5 days of antibiotics prior to diagnosis of CDAD	PPI
Muto et al,^44^ 2005	US	Single	Hospital	Gen Inpatients	Case control	Nosocomial CDAD	PPI: During the 4 weeks before detection of CDAD
Yip et al,^45^ 2001	CA	Single	Hospital	Gen Inpatients	Case control	Nosocomial CDAD	PPI
Linney et al,^41^	CA	Single	Hospital	Gen Inpatients	Case control	Nosocomial, onset during outbreak.	PPI being taken on the date of CDAD diagnosis
Linney et al,^41^	CA	Single	Hospital	Gen Inpatients	Case control	Nosocomial, onset during outbreak.	PPI being taken before the date of CDAD diagnosis

♦ NA: Data obtained from abstract

Legend: OR: odds ratio; HR: harzard ratio; CDAD: *Clostridium difficile-*associated diarrhea; PPI: Proton pump inhibitor; H2RA: VA: Veteran Affairs; D: Durham County, P: Prospective; R: Retrospective, ABX: Antibiotic; GPRD: General Practice Research Database; ICU: Intensive care unit

Metabolic processes are controlled by a variety of enzymes. For instance, cytochrome P450 monooxygenase could detoxify herbicides such as fenoxaprop-ethyl, diclofop-methyl, and bentazon in plants [22], [23]. Polyphenol oxidase (PPO), commonly found in fungi and plants, refers to a group of enzymes that catalyze the oxidation of phenolic compounds [24]. Peroxidase (POD), another type of oxidative enzyme commonly present in plant and animal tissues, can oxidize phenols and aromatic amines in the presence of hydrogen peroxide. In contrast, the oxidation of phenolic compounds by PPO requires the presence of oxygen gas [25]. Both PPO and POD play important roles in the metabolism of aromatic compounds in soil and water [26], [27]. However, little information is available regarding their function in the metabolism of PAHs by plants.

### Quality Assessment of Included Studies

Quality assessment of all included studies was done using the validated Newcastle-Ottawa Quality Assessment Scale [27] for cohort and case control studies (Table S3 and Table S4). Most studies were of good quality with no evidence of selection bias, and with good comparability of the exposed and unexposed groups of each cohort, and outcome assessment. Fifty-one individual effect estimates from 47 eligible citations were extracted. We identified 2 outliers and excluded them from the final analyses as per the Cochrane Handbook for Systematic Reviews [39]. The 2 outliers were: Bajaj et al [40] because of a high OR = 37.6, and Wilcox et al [41] because of large SE (SE log OR = 3.59). Final interpretation was based on analyses of the 51 observations.

### Meta-analysis

#### Association between PPI and CDI

**Figure 2:**
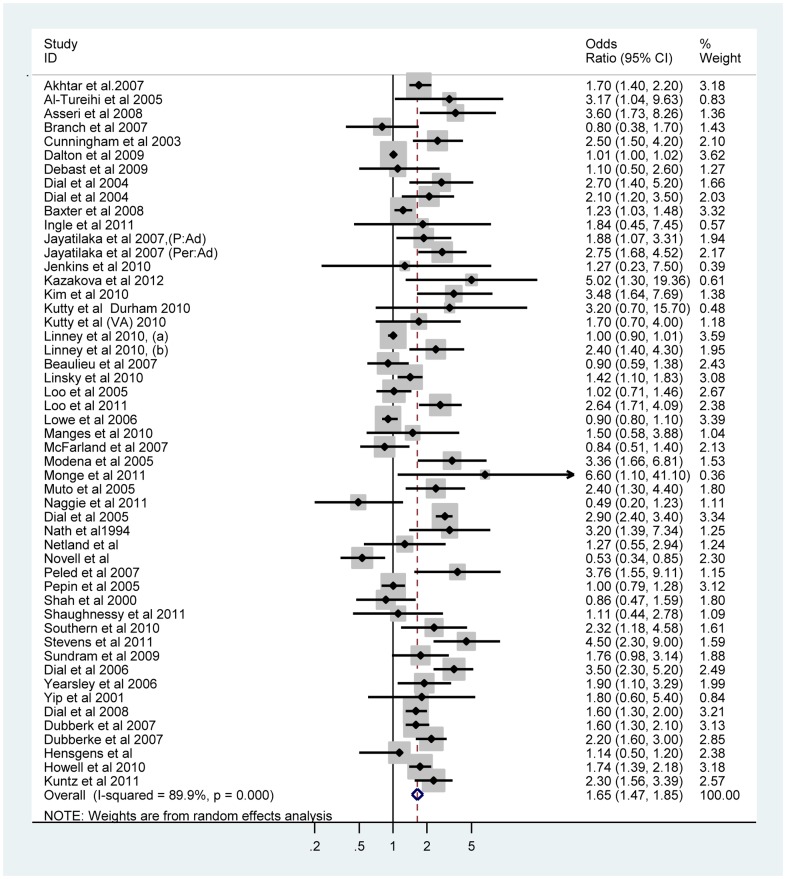
Forest Plot of the Meta analyses of The Association Between CDI and Proton Pump Inhibitors Based on 51 Observations.

Fifty one individual effect estimates from 47 eligible studies were extracted. Figure 2 shows the results of the pooled estimates for the 51 observations. The pooled OR for the 51 observations was 1.65, 95% CI (1.47, 1.85), I^2^ = 89.9%. Table 2 summarizes the pooled estimates and associated heterogeneity for different subgroups. All estimates supported an association between PPI therapy and CDI.

**Table 2:** Influence of study type, country, weather effect estimate adjusted or not and PPI ascertainment method on the pooled effect estimate and its associated heterogeneity.

Group	Pooled Effect Estimate 95% CI	I^2%^	Number of Observations
All citations	1.65 (1.47, 1.85)	89.9	51
Case-control citations	1.70 (1.42, 2.03)	88.7	37
Cohort citations	1.64 (1.30, 2.08)	87.8	14
Asia	3.26(1.91, 5.58)	0.0	3
Canada	1.22 (1.09, 1.37)	82.1	14
Europe	1.90 (1.35, 2.66)	75.5	10
USA	1.70 (1.41, 2.04)	73.3	24
Studies reported adjusted effect estimates*	1.78 (1.56, 2.02)	92.2	37
Studies reported unadjusted effect estimates*	1.27 (0.93, 1.71)	59.4	14
PPI ascertainment method (Chart)^†^	1.89 (1.45, 2.45)	93.6	20
PPI ascertainment method (Interview)^†^	1.17 (0.91, 1.51)	79.0	8

#### Exploring heterogeneity

The influence of a range of a-priori selected study-level and aggregated individual-level parameters on the observed effect estimate was investigated by means of meta-regressions. Table S5 summarizes the meta-regression analyses for all 51 results and is presented in the appendix. We observed that studies that used interviews to ascertain PPI exposure had on average lower effect estimates that studies that used medical records 1.17 (0.91, 1.51) vs. 1.89 (1.45, 2.45), p for interaction = 0.05 (Table 2). We also observed that studies that used adjusted effect estimates [1.76 (95% CI, 1.54, 2.00)] had higher pooled estimates than those that used unadjusted effect estimates [1.27 (95% CI, 0.93, 1.72)], p = 0.07.

#### Publication bias

**Figure 3:**
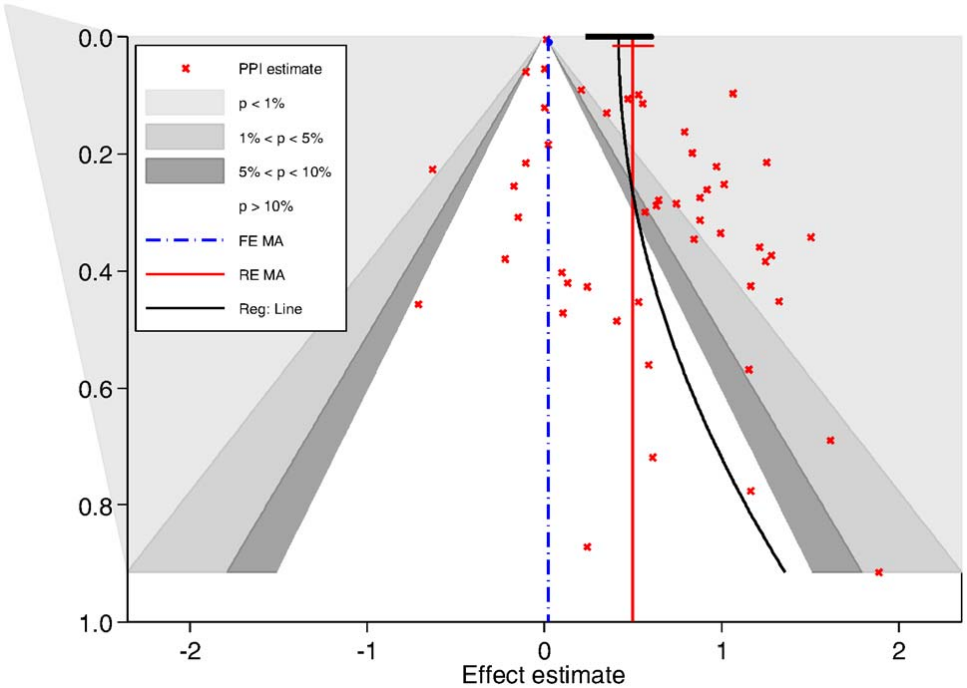
Contour enhanced funnel plot of the association between the effect-estimates and its standard errors: * Contour enhanced funnel plots with implementation of regression adjustment model (adjusted effect at top where SE is 0).* The contour lines differentiate the significance and non-significance regions in the plot at 1%, 5% and 10% significance levels. *Vertical lines show average effect-estimates from random effect (red), and fixed effect models (blue). *A regression line (black) is added for regression based adjustment (With adjusted effect estimate and 95% CI at top where SE is 0). Abbreviations: FEMA: Fixed effect meta-analysis, REMA: Random effect meta-analysis, Reg: Regression line.

Figure 3 displays a contour-enhanced funnel plot with the corresponding fixed (FE) and random effect (RE) meta-analyses pooled estimates providing a weighted average of effect size across studies of 1.02 (95% CI, 1.01–1.03) and 1.65 (95% CI, 1.47–1.85) respectively. There was visual evidence of funnel asymmetry and Egger’s test for publication bias, P = 0.001. Hence, a novel regression based method was used to adjust for publication bias.

The fitted regression line plotted in Figure 3 corresponds to the regression-based adjustment method. The adjusted estimate is obtained by extrapolating the line with a standard error of 0 (at the top of the funnel plot). This produced an adjusted average effect estimate (RE model) of 1.51 (95% CI, 1.26–1.83).

#### Residual confounding

**Figure 4:**
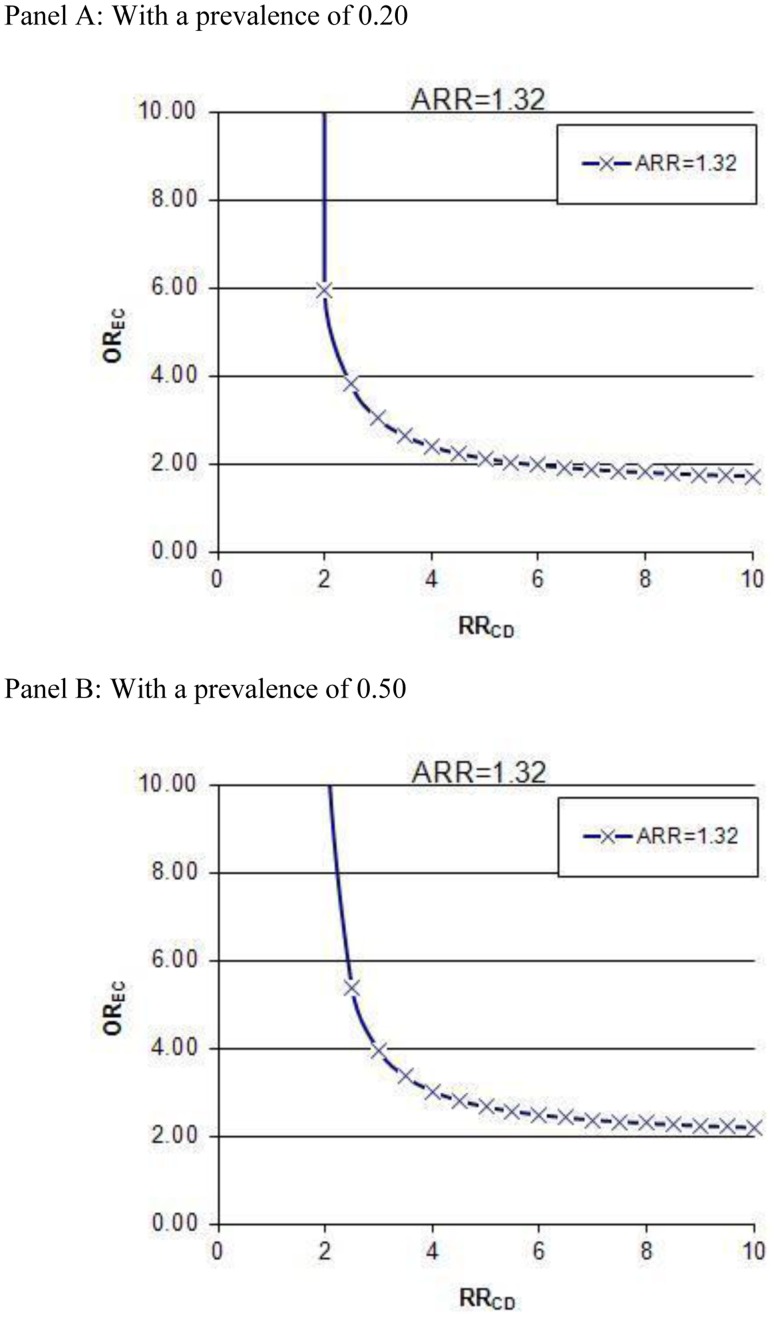
Influence of a hypothetical dichotomous confounder present in 20% (panel A) and 50% (panel B) of the study population, unaccounted for in the adjustments already performed in the individual studies. The graphs indicate what combinations of OREC and RR that would be necessary for the confounder to fully account for the observed association between proton pump inhibitor (PPI) use and CDAD after adjustment for publication bias. Abbreviations: OR_EC_, odds ratio of exposure to the confounder in PPI non-users vs. acid-suppression users; RR_CD_, relative risk of CDAD in individuals exposed to the confounder vs. non-exposed.

The results of the residual confounding analysis are presented in Figure 4. Panel A refers to a confounder with a prevalence of 0.20 and at this prevalence level, even a strong confounder causing a 2.5-fold increased risk of CDI would have to be imbalanced between acid-suppression users and non-users (OREC = 3.8) to fully account for the observed adjusted RR of 1.32 (adjusted for publication bias). For a very common confounder with a prevalence of 0.50 (Panel B) and causing a 2.5-fold increased risk of CDI, it would have to be distinctly imbalanced between acid-suppression users and non-users (OREC = 5.38) to fully account for the observed adjusted RR of 1.32.

### Number Needed to Harm

The number needed to harm (NNH) was estimated by using the pooled OR from the meta-analysis [42]. This analysis is only speculative as it assumes there is a cause-effect relationship between PPI and CDI. A recent large prospective hospital cohort [43] reported the incidence of CDI at 14 days after hospital admission in patients receiving antibiotics or not: which was 42/1,000 and 5.4/1000, respectively. Based on these reported baseline risks, the number needed to harm (NNH) was 50, 95% CI (31, 97) and 367, 95% CI (226, 718), respectively. For the general population, the NNH at 1 year was 3925, 95% CI (2412, 7698) at 1 year, based on a baseline incidence of CDI of 48/100,000 person-years [39].

## Discussion

### Findings

In this rigorously conducted systemic review and meta-analysis, we observed a weak association between PPI use and risk of CDI. This association was further weakened by the presence of significant heterogeneity. Although we adjusted for publication bias and ruled out a strong effect of an unmeasured confounder, the cumulative evidence provided by this systematic review constitutes only very low quality evidence (as per GRADE framework) in favor of this association. Factors that negatively influence the quality of the evidence include the observational design, inconsistency of results, and evidence of publication bias.

Moreover, *even if we assume that the pooled effect estimate is valid*, the absolute risk of CDI would be very low in the general population with an estimated NNH of 3925 at 1 year. In contrast, the risk would behighest in hospitalized patients receiving antibiotics with an estimated NNH of 50 at 2 weeks.

### Comparison to Other Studies

Several systematic reviews [15], [21]–[23], [44], [45] examining this association have been published previously; however, our review is the most comprehensive and is unique in its analytical approach and interpretation and thus adds substantially to the cumulative evidence. Table 3 summarizes the differences between our systematic and those recently published. First, our review identified the largest number of studies published to date. For example, our review has 36% more studies than the largest meta-analysis by Kowk et al [21] and 90% more studies that the recent FDA review published in February 2012. Second, our meta-analysis included adjusted effect estimates of the association between PPI and CDI. Third, we used meta-regression to explore sources of heterogeneity. None of the published analyses used this method. Fourth, we examined the effect of publication bias using a novel approach of contour-enhanced funnel plot [46]. The largest and most recent analysis by Kowk et al. [21] did not examine the effect of publication bias. Fifth, we used a novel regression-based method to adjust the pooled estimate for publication bias and we examined the potential effect of a residual confounding on the observed association using the rule-out approach.

**Table 3:** Summary of reviews of the association between PPI use and *Clostridium Difficile* infection.

Study	Search Engines	Date	Acid suppression	Number of observations	Pooled effect estimates	Heterogeneity	Exploring heterogeneity	Publication bias assessment	Residual confounding
Tleyjeh et al (Present review)	Medline EMBASE ISI Web of Science and Elsevier Scopus	Jan 2012	PPI	51	1.65 (1.47, 1.85)	I^2^ = 89.9%	Meta-regression	Contour-enhanced funnel plot asymmetry	Rule-out approach
FDA Alert, ^44^	Not available	NR	PPI	28	Systematic review only No meta-analysis	NA	NA	NA	NA
Kwok et al,^21^ 2012	Medline EMBASE	Dec 2011	PPI	39	1.74 (1.47–2.85)	I^2^ = 85%	Sensitivity analyses: -Case-control -Cohort -Lab. Confirmation -Inpatient -Community or mixed -Adjusted estimates	Not assessed	Not assessed
Bavishi and Dupont, ^23^ 2011	Medline	May 2011	PPI	27	Systematic review only No meta-analysis	NA	NA	NA	NA
Janarthanan et al,^45^ 2012	Medline	Dec 2010	PPI	23	1.69 (1.395–1.974)	I^2^ = 91.9%	Subgroup analysis: Case-control -Cohort	Funnel plot: possible asymmetry	Not assessed
Deshpande et al,^22^ 2012	Medline CINAHL Cochrane Web of Science and Scopus	Oct 2010	PPI	30	2.15 (1.81–2.55) **Unadjusted data**	I^2^ = 87%	-Case-control -Cohort -% antibiotic used	Funnel plot: no asymmetry	Not assessed
Leonard et al,^15^ 2007	Medline CINAHL EMBASED	2005	PPI	11	2.05 (1.47–2.85) **Unadjusted data**	P<0.001	Not assessed	Funnel plot: no asymmetry	Not assessed

Legend: PPI: Proton pump inhibitor; NA: Not available.

Finally, we interpret the results in the context of observed limitations and therefore, draw more careful conclusions. Contrary to other reviews and FDA alert, we conclude that current cumulative data constitutes very low quality evidence. Our results are helpful for guidelines writing committees and policy makers that use the GRADE framework when formulating recommendations for use of PPI for different clinical indications.

### Biologic Plausibility

The mechanism by which PPI therapy contributes to an increased risk of CDI is unclear, because gastric acid does not kill gastric *C. difficile* spores. This further makes a cause-effect relationship a less likely explanation for the observed association.

It has been proposed that the vegetative form of *C. difficile*, which is killed by acid, plays a role in pathogenesis. Vegetative forms survive on surfaces and could be ingested by patients [47]. Survival of these acid-sensitive vegetative forms in the stomach could be facilitated by 2 main factors: (1) suppression of gastric acid production by acid-suppressive medications; and (2) presence of bile salts in gastric contents of patients on acid-suppressive therapy. Bile salts, which are mainly found in the small intestine, are present in gastric contents, particularly among patients with gastro-esophageal reflux disease (GERD). Moreover, PPI use can delay gastric emptying and predispose to bacterial overgrowth with associated high intragastric bile salts which could trigger spore germination in the stomach [48]–[50].

However, a recent in vitro experiment has challenged these postulated biological mechanisms for the observed association. In this experiment, aspirate of gastric contents from hospitalized patients with nasogastric tubes were collected. It concluded that *C. difficile* spores were not killed in acidic gastric content and did not germinate in gastric contents of hospitalized patients on PPI. Germination occurred with the addition of taurocholic acid [51].

### Limitations

There are limitations to our work. First, observational studies are subject to inherent limitations in the study design leading to unmeasured differences in the study population and unmeasured confounders despite all possible adjustments. PPIs use may be a surrogate of comorbidities and thus, the observed association may have been affected by selective overuse of PPIs in high risk groups. For example, the potential interaction between PPIs and Clopidogrel found in observational studies was refuted in randomized controlled trials [52]. Second, the use of PPIs was based on electronic and prescription records, rather than by actual use by the patient. Third, there is presence of publication bias, and substantial amount of heterogeneity in the included studies. There are many patient level parameters which may have led to substantial heterogeneity. Nevertheless, investigating these variables is only possible with individual patient data meta-analysis. Fourth, all statistical methods used to assess for publication bias or residual confounding are subject to certain assumptions and have inherent limitations. For example, funnel plot asymmetry can be due to between studies heterogeneity rather than publication bias [31].

Given these limitations, focus on hand hygiene as one of the cornerstones of prevention of nosocomial transmission of *C. difficile* is warranted. Several studies have documented the reduction of rates of hospital acquired infection by improvement in the compliance with hand washing by healthcare workers between episodes of contact with patients [53].

### Conclusions

In this rigorously conducted systemic review and meta-analysis, we found very low quality evidence in support of an association between PPI use and risk of CDI. This association was weakened by the presence of significant heterogeneity and publication bias. Our findings are re-assuring that PPIs use in the general population does not pose a significant CDI risk. On the other hand, our findings warrant judicious and evidence-based use of PPI in patients at high risk for CDI.

## Supporting Information

Table S1 **The Association between PPI use and Development of** ***Clostridium difficile*** **infection in Case-control Citations.**
(DOCX)

Table S2 **The Association between PPI use and Development of** ***Clostridium difficile*** **infection from Cohort citations.**
(DOCX)

Table S3 **Meta-regression analysis to explore sources of heterogeneity.**
(DOCX)

Table S4 **Forest plot of the meta-analysis of the proportion of** ***Clostridium difficile*** **cases that were exposed to antibiotics.**
(DOCX)

Table S5 **Modified Newcastle-Ottawa Quality Assessment Scale for Case-control studies included in the Meta-analysis.**
(DOCX)

Figure S1 **Forest plot of the meta-analysis of the proportion of** ***Clostridium difficile*** **cases that were exposed to antibiotics.**
(DOCX)

## References

[1] IMS Health (2005) Leading 20 therapeutic classes by U.S. sales, Available: http: //www.imshealth.com/ims/portal/front/articleC/0,2777,6599_73915261_77140565,00.html. Accessed 2006 June.

[2] Yachimski PS, Farrell EA, Hunt DP, Reid AE (2010) Proton Pump Inhibitors for Prophylaxis of Nosocomial Upper Gastrointestinal Tract BleedingEffect of Standardized Guidelines on Prescribing Practice. Arch Intern Med. 2010 170(9): 779–783. 10.1001/archinternmed.2010.51 PMC3758922 20458085

[3] Batuwitage BT, Kingham JGC, Morgan NE, Bartlett RL (2007) Inappropriate prescribing of proton pump inhibitors in primary care. Postgrad Med J 83: 66–68. 17267683 10.1136/pgmj.2006.051151 PMC2599965

[4] Cahir C, Fahey T, Teeling M, Teljeur C, Feely J, et al (2010) Potentially inappropriate prescribing and cost outcomes for older people: a national population study. Br J Clin Pharmacol 69(5): 543–552. 20573091 10.1111/j.1365-2125.2010.03628.x PMC2856056

[5] Mat Saad AZ, Collins N, Lobo MM, O’Connor HJ (2005) Proton pump inhibitors: a survey of prescribing in an Irish general hospital. Int J Clin Pract 59: 31–34. 15707461 10.1111/j.1742-1241.2004.00298.x

[6] Niklasson A, Bajor A, Bergendal L, Simren M, Strid H, et al (2003) Overuse of acid suppressive therapy in hospitalized patients with pulmonary diseases. Respir Med 97: 1143–1150. 14561022 10.1016/s0954-6111(03)00187-2

[7] Scagliarini R, Magnani E, Pratico A, Bocchini R, Sambo P, et al (2005) Inadequate use of acid-suppressive therapy in hospitalized patients and its implications for general practice. Dig Dis Sci 50: 2307–2311. 16416179 10.1007/s10620-005-3052-4

[8] Ahrens D, Chenot JF, Behrens G, Grimmsmann T, Kochen MM (2010) Appropriateness of treatment recommendations for PPI in hospital discharge letters. Eur J Clin Pharmacol 66(12): 1265–1271. 20694459 10.1007/s00228-010-0871-9 PMC2982961

[9] US Food and Drug Administration (2009) Information for healthcare professionals: update to the labeling of clopidogrel bisulfate (marketed as Plavix) to alert healthcare professionals about a drug interaction with omeprazole (marketed as Prilosec and Prilosec OTC). U.S. Department of Health and Human Services, 11/17/2009. http: //www.fda.gov/Drugs/Drug Safety/Postmarket Drug Safety Information for Patients and Providers/Drug Safety Information for Heath,care Professionals/ucm190787.htm. Accessed 9/23/2010.

[10] Charlot M, Grove EL, Hansen PR, Olesen JB, Ahlehoff O, et al (2011) Proton pump inhibitor use and risk of adverse cardiovascular events in aspirin treated patients with first time myocardial infarction: nation-wide propensity score-matched study. BMJ 342: d2690. 21562004 10.1136/bmj.d2690 PMC3092520

[11] Eom CS, Park SM, Myung SK, Yun JM, Ahn JS (2011) Use of acid suppressive drugs and risk of fracture: a meta-analysis of observational studies. Ann Fam Med 9(3): 257–267. 21555754 10.1370/afm.1243 PMC3090435

[12] Sierra F, Suarez M, Rey M, Vela MF (2007) Systematic review: proton pump inhibitor-associated acute interstitial nephritis. Aliment Pharmacol Ther 26: 545–553. 17661758 10.1111/j.1365-2036.2007.03407.x

[13] Johnstone J, Nerenberg K, Loeb M (2010) Meta-analysis: proton pump inhibitor use and the risk of community-acquired pneumonia. Aliment Pharmacol Ther 31(11): 1165–1177. 20222914 10.1111/j.1365-2036.2010.04284.x

[14] Dial MS. (2009) Proton pump inhibitor use and enteric infections. Am J Gastroenterol (suppl 2): S10–S16. 10.1038/ajg.2009.46 19262540

[15] Leonard J, Marshall JK, Moayyedi P (2007) Systematic review of the risk of enteric infection in patients taking acid suppression. Am J Gastroenterol 102: 2047–2056. 17509031 10.1111/j.1572-0241.2007.01275.x

[16] Jarvis WR, Schlosser J, Jarvis AA, Chinn RY (2009) National point prevalence of Clostridium difficile in US health care facility inpatients, 2008. Am J Infect Control 37: 263–270. 19278754 10.1016/j.ajic.2009.01.001

[17] Archibald LK, Banerjee SN, Jarvis WR (2004) Secular trends in hospital-acquired Clostridium difficile disease in the United States. J Infect Dis 189: 1585–1589. 15116293 10.1086/383045

[18] McDonald LC, Owings M, Jernigan JB (2006) *Clostridium difficile* infection in patients discharged from US short-stay hospitals, 1996–2003. Emerg Infect Dis 12: 409–415. 16704777 10.3201/eid1203.051064 PMC3291455

[19] Elixhauser A, Jhung M (2008) *Clostridium difficile*-associated disease in US hospitals, 1993–2005. HCUP statistical brief No. 50, April 2008. US Agency for Healthcare Research and Quality, Rockville, MD. Available at: http: //www.hcup-us.ahrq.gov/reports/statbriefs.sb50.pdf. Accessed February 16, 2009.

[20] Redelings MD, Sorvillo F, Mascola L (2007) Increase in *Clostridium difficile*-related mortality rates, United States, 1999e2004. Emerg Infect Dis 13(9): 1417–1419. 18252127 10.3201/eid1309.061116 PMC2857309

[21] Kwok CS, Arthur AK, Anibueze CI, Singh S, Cavallazzi R, et al (2012) Risk of *Clostridium difficile* Infection With Acid Suppressing Drugs and Antibiotics: Meta-Analysis. Am J Gastroenterol 107(7): 1011–1019. 22525304 10.1038/ajg.2012.108

[22] Deshpande A, Pant C, Pasupuleti V, Rolston DD, Jain A, et al (2012) *Association bet*ween proton pump inhibitor therapy and *Clostridium difficile* infection in a meta-analysis. Clin Gastroenterol Hepatol 10(3): 225–233. 22019794 10.1016/j.cgh.2011.09.030

[23] Bavishi C, Dupont HL (2011) Systematic review: the use of proton pump inhibitors and increased susceptibility to enteric infection. Aliment Pharmacol Ther 34(11–12): 1269–1281. 21999643 10.1111/j.1365-2036.2011.04874.x

[24] Stroup DF, Berlin JA, Morton SC, Olkin I, Williamson GD, et al (2000) Meta-analysis of Observational Studies in Epidemiology: A Proposal for Reporting. JAMA 283: 2008–2012. 10789670 10.1001/jama.283.15.2008

[25] Liberati A, Altman DG, Tetzlaff J, Mulrow C, Gøtzsche PC, et al (2009) The PRISMA statement for reporting systematic reviews and meta-analyses of studies that evaluate healthcare interventions: explanation and elaboration. *BMJ* 2009 339: b2700. 10.1136/bmj.b2700 PMC2714672 19622552

[26] Guyatt GH, Oxman AD, Vist G, Kunz R, Falck-Ytter Y, et al (2008) Rating quality of evidence and strength of recommendations GRADE: an emerging consensus on rating quality of evidence and strength of recommendations. BMJ 336: 924–926. 18436948 10.1136/bmj.39489.470347.AD PMC2335261

[27] Wells G, Shea B, O’Connell D, Peterson J, Welch V et al. The Newcastle- Ottawa scale (NOS) for assessing the quality of non-randomized studies in meta-analysis. Ottawa, Ontario: The Ottawa Health Research Institute. Available: http: //www.ohri.ca/programs/clinicalepidemiology/nosgen.doc. Accessed on 2011 September 13.

[28] Higgins JPT, Altman DG (2008) Chapter 8: Assessing risk of bias in included studies. In: Higgins JPT, Green S, editors. Cochrane Handbook for Systematic Reviews of Interventions Version 5.0.1 [updated September 2008] The Cochrane Collaboration, 2008. Available: www.cochrane-handbook.org. Accessed 2010 December 15.

[29] DerSimonian R, Laird N (1986) Meta-analysis in clinical trials. Control Clin Trials 7(3): 177–188. 3802833 10.1016/0197-2456(86)90046-2

[30] Peters JL, Sutton AJ, Jones DR, Abrams KR, Rushton L (2008) Contour-enhanced meta-analysis funnel plots help distinguishing publications bias from other causes of asymmetry. J Clin Epidemiol 61: 991–996. 18538991 10.1016/j.jclinepi.2007.11.010

[31] Egger M, Davey Smith G, Schneider M, Minder C (1997) Bias in meta-analysis detected by a simple, graphical test. BMJ 315: 629–634. 9310563 10.1136/bmj.315.7109.629 PMC2127453

[32] Moreno SG, Sutton AJ, Ades AE, Stanley TD, Abrams KR, et al (2009) Assessment of regression-based methods to adjust for publication bias through a comprehensive simulation study. BMC Med Res Methodol 9: 2. 19138428 10.1186/1471-2288-9-2 PMC2649158

[33] Schneeweiss S (2006) Sensitivity analysis and external adjustment for unmeasured confounders in epidemiologic database studies of therapeutics. Pharmacoepidemiol Drug Saf 15: 291–303. 16447304 10.1002/pds.1200

[34] Zhang J, Yu KF (1998) What’s a relative risk? A method of correcting the odds ratio in cohort studies of common outcomes. JAMA 280: 1690–1691. 9832001 10.1001/jama.280.19.1690

[35] Dial S, Alrasadi K, Manoukian C, Huang A, Menzies D (2004) Risk of *Clostridium difficile* diarrhea among hospital inp*a*tients prescribed proton pump inhibitors: cohort and case-control studies. CMAJ 171(1): 33–38. 15238493 10.1503/cmaj.1040876 PMC437681

[36] Kutty PK, Woods CW, Sena AC (2010) Risk Factors for and Estimated Incidence of Community-associated *Clostridium difficile* Infection, North Carolina, USA. Emerg Infect Dis 16(2): 197–204. 20113547 10.3201/eid1602.090953 PMC2958012

[37] Jayatilaka S, Shakor R, Eddi R, Bakaj G, Baddoura WJ, et al (2007) *Clostridium difficile* Infection in an Urban Medical Centre: Five-year Analysis of Infection Rates among Adults admissions and Association with the Use of Protein Pump Inhibitors. Ann Clin Lab Sci 37(3): 241–247. 17709687

[38] Linney S, Fernandes T, Einarson T, Sengar A, Walker JH, et al (2010) Association Between Use of Proton Pump Inhibitors and a *Clostridium difficile*–Associated Disease Outbreak: Case–Control Study. Can J Hosp Pharm 63(1): 31–37. 22478951 10.4212/cjhp.v63i1.866 PMC2832569

[39] http: //www.cochrane-net.org/openlearning/html/mod14-2.htm.

[40] Bajaj JS, Ananthakrishnan AN, Hafeezullah M, Zadvornova Y, Dye A, et al (2010) *Clostridium difficile* is Associated With Poor Outcomes in Patients With Cirrhosis: A National and Tertiary Center Perspective. Am J Gastroenterol 105: 106–113. 19844204 10.1038/ajg.2009.615

[41] Wilcox CM, Martin T, Phadnis M, Mohnen J, Worthington J, et al (2008) Absence of gastrointestinal infections in a cohort of patients with Zollinger-Ellison syndrome and other acid hypersecretors receiving lon*g*-term acid suppression with lansoprazole. BMC Gastroenterol 8: 18. 18507843 10.1186/1471-230X-8-18 PMC2414526

[42] Lowe DO, Mamdani MM, Kopp A, Low DE, Juurlink DN (2006) Proton pump inhibitors and hospitalization for *Clostridium difficile*–associated disease: a population-based study. Clin Infect Dis 43(10): 1272–1276. 17051491 10.1086/508453

[43] Howell MD, Novack V, Grgurich P, Soulliard D, Novack L, et al (2010) Iatrogenic Gastric Acid Suppression and the Risk of Nosocomial Clostridium difficile Infection. Arch Intern Med 170(9): 784–790. 20458086 10.1001/archinternmed.2010.89

[44] Available: http: //www.fda.gov/Drugs/DrugSafety/ucm290510.htm.Accessed 2012 February 14.

[45] Janarthanan S, Ditah I, Adler DG, Ehrinpreis MN (2012) *Clostridium difficile*-Associated Diarrhea and Proton Pump Inhibitor Therapy: A Meta-Analysis. Am J Gastroenterol 107(7): 1001–1010. 22710578 10.1038/ajg.2012.179

[46] Moreno SG, Sutton AJ, Turner EH, Abrams KR, Cooper NJ, et al (2009) Novel methods to deal with publication biases: secondary analysis of antidepressant trials in the FDA trial registry database and related journal publications. BMJ 339: b2981. 19666685 10.1136/bmj.b2981 PMC2723217

[47] Jump RL, Pultz MJ, Donskey CJ (2007) Vegetative *Clostridium difficile* survives in room air on moist surfaces and in gastric contents with reduced acidity: a potential mechanism to explain the association between proton pump inhibitors and *C. difficile*-associated diarrhea? Antimicrob Agents Chemother 51: 2883–2887. 17562803 10.1128/AAC.01443-06 PMC1932506

[48] Wilson KH (1983) Efficacy of various bile salt preparations for stimulation of *Clostridium difficile* spore germination. J Clin Microbiol 18: 1017–1019. 6630458 10.1128/jcm.18.4.1017-1019.1983 PMC270959

[49] Theisen J, Nehra D, Citron D, Johannson J, Hagen J, et al (2000) Suppression of gastric acid secretion in patients with gastroesophageal reflux disease results in gastric bacterial overgrowth and deconjugation of bile acids. J Gastrointest Surg 4: 50–54. 10631362 10.1016/s1091-255x(00)80032-3

[50] Thorens J, Froehlich F, Schwizer W, Saraga E, Bille J, et al (1996) Bacterial overgrowth during treatment with omeprazole compared with cimetidine: a prospective randomized double blind study. Gut 39: 54–59. 8881809 10.1136/gut.39.1.54 PMC1383231

[51] Nerandzic MM, Pultz MJ, Donskey CJ (2009) Examination of Potential Mechanisms To Explain the Association between Proton Pump Inhibitors and *Clostridium difficile* Infection. Antimicrob Agents Chemother 53(10): 4133–4137. 19667292 10.1128/AAC.00252-09 PMC2764230

[52] Bhatt DL, Cryer BL, Contant CF, Cohen M, Lanas A, et al (2010) Clopidogrel with or without omeprazole in coronary artery disease. N Engl J Med 363: 1909–1917. 20925534 10.1056/NEJMoa1007964

[53] Boyce JM, Pittet D (2002) Healthcare Infection Control Practices Advisory Committee; HICPAC/SHEA/APIC/IDSA Hand Hygiene Task Force. MMWR Recomm Rep. 25 51(RR-16): 1–45. 12418624

