# Supplementary material for: Association between Proton Pump Inhibitor Therapy and Clostridium difficile Infection: A Contemporary Systematic Review and Meta-Analysis
Source: PLoS One. 2012 Dec 7;7(12):e50836. doi: 10.1371/journal.pone.0050836 (PMC3517572; doi:10.1371/journal.pone.0050836)
Supplement: Table S1 — The Association between PPI use and Development of Clostridium difficile infection in Case-control Citations. (DOCX) [file pone.0050836.s001.docx]

| **Table S1. The Association between PPI use and Development of *Clostridium Difficile* infection in Case-control Citations** | | | | |
| --- | --- | --- | --- | --- |
| **Adjusted Effect Estimates** | **Sample Size** | **Selection of Controls** | **Case Ascertainment** | **Source** |
| Crude OR, 1.7 CI (0.7–4.0) | Exposed group; cases: 13, controls: 26  Non-exposed group; cases:23, controls: 82 | Random selection from same  outpatient geographical location | Non-formed stool  Stool positive for CD toxin | Kutty et al (VA),^1^ 2010 |
| Crude OR, 3.2 CI (0.7–15.7) | Exposed group; cases: 09, controls: 2  Non-exposed group; cases:64, controls: 46 | Random selection from same  outpatient geographical location | Non-formed stool,  Stool positive for CD toxin | Kutty et al (D),^1^ 2010 |
| OR 2.32, CI (1.18-4.58) | Exposed group; cases: 13, controls: 391  Non-exposed group; cases: 38, controls: 3513 | Post abdominal surgery, operated at  the same institutions during the same time | Diarrhea; Positive stool for CD within 30d post abdominal surgery | Southern et al,^2^ 2010 |
| OR 3.2, CI (1.39-7.34) | Exposed group; cases: 51, controls: 32  Non-exposed group; cases:29, controls: 48 | Age matched, Same hospital unit | Diarrhea  Positive stool for CD toxin | Nath et al,^3^ 1994 |
| OR : 2.75, CI ( 1.68 – 4.52) | Exposed group; cases: 84, controls: 112  Non-exposed group; cases: 38, controls: 132 | Age and sex matched, Same period of time. | Pre or during admission use of PPI:  Diarrhea, Positive stool for CD toxins | Jayatilaka et al,^4^ 2007 |
| 1.88, CI ( 1.07-3.31) | Exposed group; cases: 34, controls: 46  Non-exposed group; cases: 88, controls: 198 | Age and sex matched, Same period of time. | Post admission use of PPI:  Diarrhea, Positive stool for CD toxins | Jayatilaka et al,^4^ 2007 |
| Crude OR : 0.79, P value : 0.442 | Exposed group; cases: 34, controls: 37  Non-exposed group; cases: 23, controls: 20 | Matched to Age, Sex, Dialysis, TPN, H_2_RAs, same period of hospitalization. | Diarrhea  Positive stool for CD toxin | Branch et al,^5^ 2007 |
| Crude OR : 3.1;  p < .05 | Exposed group; cases: 15, controls: 09  Non-exposed group; cases: 10, controls: 19 | Treated with antibiotics other than Metronidazole and Vancomycin, Matched by Age/antibiotics treatment. | Positive stool for CD toxins, Diagnosis/antibiotic for CDI, | Al-Tureihi et al,^6^ 2005 |
| OR :1.23, CI (1.03-1.48) | Exposed group; cases: 457, controls: 939  Non-exposed group; cases: 685, controls: 2412 | Matched to LOS, Hospitalized in the same hospital during the same period. | Diarrhea  Positive stool for CD toxin | Baxter et al,^7^ 2008 |
| Crude OR : 0.86, P value : 0.35 | Exposed group; cases: 24, controls: 27  Non-exposed group; cases: 102, controls: 99 | Negative stool toxins, Similar age, Hospital ward, Same time | Diarrhea  Positive stool for CD toxin | Shah et al,^8^ 2000 |
| PPI ≤ 90d,  OR: 0.9 (0.8-1.1)  PPI 91-180d,  OR: 0.7 (0.5-1.0)  PPI 181-365d,  OR: 0.9 (0.6-1.3) | PPI ≤ 90d  Exposed group; cases: 306, controls: 2254  Non-exposed gp; cases: 1083, controls: 10049  PPI 91-180d  Exposed group; cases: 30, controls: 331  Non-exposed gp; cases: 1359, controls: 11972  PPI 181-365  Exposed group; cases: 37, controls: 317  Non-exposed group; cases: 102, controls: 99 | Matched to age, sex, and antibiotic use | CDAD | Lowe et al,^9^ 2006 |
| OR : 1.90, (1.10-3.29) | Exposed group; cases: 62, controls: 38  Non-exposed group; cases: 93, controls: 115 | Matched to age , From the same ward at the time of recruitment | Diarrhea  Positive stool for CD toxin | Yearsley et al,^10^ 2006 |
| OR : 2.9, (2.4-3.4) | Exposed group; cases: 280, controls: 1038  Non-exposed gp; cases: 953, controls: 11292 | same general practice, Not hospitalized in the year prior to index date, Negative CD toxin, No diagnosis of CDI | Positive CD toxin  clinical diagnosis made by GP | Dial et al,^11^ 2005 |
| OR : 1.7 (1.4-2.2) | Exposed group; cases: 274, controls: 169  Non-exposed group; cases: 366, controls: 481 | Age, sex matched;, Diarrhea with negative CD | Diarrhea  Positive CD assay | Akhtar et al,^12^ 2007 |
| OR : 3.5 (2.3-5.2) | Exposed group; cases: 61, controls: 157  Non-exposed group; cases: 256, controls: 2010 | Age matched, Same ward | Patients with first prescription of oral Vancomycin | Dial et al,^13^ 2006 |
| OR : 3.6, (1.73-8.26) | Exposed group; cases: 61, controls: 36  Non-exposed group; cases: 33, controls: 58 | Matched to date of admission, antibiotic use, gender, age group, patient location, room type | Diarrhea  Positive stool for CD toxin | Asseri et al,^14^ 2008 |
| OR : 2.5, (1.5-4.2) | Exposed group; cases: 58, controls: 31  Non-exposed group; cases: 102, controls: 129 | Matched to Age, sex, admitting consultant, month of admission, diagnostic code. | Positive for CD toxin | Cunningham et al,^15^ 2003 |
| OR : 2.2, (1.6-3.0) | Exposed group; cases: 267, controls: 552  Non-exposed group; cases: 115, controls: 976 | Randomly selected During the study period | Diarrhea  Positive stool for CD toxin | Dubberke et al,^16^ 2007 |
| OR :2.7, (1.4-5.2) | Exposed group; cases: 60, controls: 34  Non-exposed group; cases: 34, controls: 60 | Pharmacy records, received antibiotics, matched by age, ward, class of antibiotics | Diarrhea  Positive stool for CD toxin | Dial et al,^17^ 2004 |
| Crude OR : 1, P value : 0.92 | Exposed group; cases: 112, controls: 111  Non-exposed group; cases: 125, controls: 126 | Matched to Age, Charlson index, date of admission, ward, LOS | Diarrhea/positive CD, Endoscopic diagnosis, histological evidence | Loo et al,^18^ 2005 |
| Crude OR for PPI/ H_2_RAs : 1.7, P 0.456 | Exposed group; cases: 65, controls: 52  Non-exposed group; cases: 32, controls: 45 | Inpatients, No diarrhea, Never tested positive for CD | Diarrhea , Positive stool for CD toxin, ribotyped | Sundram et al,^19^ 2009 |
| OR 1.1, (0.5–2.6) | Exposed group; cases: 21, controls: 31  Non-exposed group; cases: 24, controls: 51 | Randomly selected from the same time and same wards as CDI cases | Diarrhea  Positive stool for CD toxin | Debast et al,^20^ 2009 |
| OR 0.53, P value = 0.01 | Exposed group; cases: 109, controls: 132  Non-exposed group; cases: 65, controls: 42 | Matched to inpatient unit, age , sex , date of admission. | New diarrhea, Positive stool for CD toxins, No other cause for the diarrhea | Novell and Moreale,^21^ 2010 |
| OR=1.14, CI(0.51-2.58) | Exposed group; cases: 64, controls: 38  Non-exposed group; cases: 29, controls: 38 | Matched for the ward and time of admission | Diarrhea, Positive C.D toxin | Hensgens et al,^22^  2011 |
| OR=1.26, P Value=1 | Exposed group; cases: 2, controls: 5  Non-exposed group; cases: 6, controls: 19 | Matched to surgeon, age, operation | Diarrhea, Positive CD toxin | Jenkins et al,^23^  2010 |
| Crude OR=1.49, P Value=0.53 | Exposed group; cases: 13, controls: 21  Non-exposed group; cases: 12, controls: 29 | Matched to age, sex, date of hospitalization | Diarrhea and: Positive CD toxin or Pseudomembranous colitis or Histological diagnosis | Manges et al,^24^ 2010 |
| OR=0.49, CI(0.2-1.23), P Value=0.13 | Exposed group; cases: 22, controls: 44  Non-exposed group; cases: 44, controls: 70 | Matched by geographic location | Diarreha  Positive CD toxin | Naggie et al,^25^ 2011 |
| OR=2.30, CI ( 1.56-3.39) | Exposed group; cases: 55, controls: 157  Non-exposed group; cases: 249, controls: 2883 | Randomly selected | ICD-9 code 008.45 for ‘Infection due to Clostridium difficile | Kuntz et al,^26^ 2011 |
| OR=6.6, CI(1.1-41.1), P Value=0.043 | Exposed group; cases: 31, controls: 43  Non-exposed group; cases: 7, controls: 33 | Matched by age, gender, admission date | Diarrhea and: Positive CD toxin or pseudomembranous colitis or toxic megacolon | Monge et al,^27^ 2011 |
|  |  |  |  |  |
| RR:1.60 (1.30.2.00) | NR | Randomly selected, matched to index date and date of first hospital admission | ICD-9code 008.45, CDAD | Dial et al,^28^ 2008 |
|  |  |  |  |  |
| NR | Exposed group; cases: 34, controls: 150  Non-exposed group; cases: 39, controls: 145 | Matched to time of CDAD, Age, Ward | Acute diarrhea  Culture positive or positive C.D toxins  No other cause for the diarrhea | McFarland et al,^29^ 2007 |
| OR:5.02(1.30-19.36) | Exposed group; cases:19, controls: 49  Non-exposed group; cases: 13, controls: 114 | Matched to Sex, Age, admission date | Diarrhea , positive CD toxin A | Kazakova et al,^30^ 2012 |
| OR:3.36(1.66-6.81) | Exposed group; cases:38, controls:12  Non-exposed group; cases: 97, controls: 103 | Inpatients , Received antibiotics for at least 5 days | Diarrhea  Positive stool for CD toxins | Modena et al,^31^ 2005 |
| OR:2.40 (1.30-4.40) | Exposed group; cases:78, controls:125  Non-exposed group; cases: 54, controls: 149 | Matched to admission date, Type of medical service, Length of hospital stay | Diarrhea  Positive stool for CD toxin | Muto et al,^32^ 2005 |
| OR:1.80(0.6-5.40) | Exposed group; cases:9, controls:18  Non-exposed group; cases: 5, controls: 22 | Matched to Age, Gender, admission date | Diarrhea  Positive stool for CD toxin | Yip et al,^33^ 2001 |
|  |  |  |  |  |
| OR:1.0(0.9-1.01) | Exposed group; cases:69, controls:73  Non-exposed group; cases: 69, controls: 73 | Controls were matched to  sex, date of discharge ,  hospital unit | Past PPI use  Positive stool for CD toxin | Linney et al,^34^ 2010, |
| OR:2.4(1.4-4.3) | Exposed group; cases:51, controls:91  Non-exposed group; cases: 34, controls: 108 | Controls were matched to  sex, date of discharge ,  hospital | Past PPI use  Positive stool for CD toxin | Linney et al,^34^ 2010, |

Legend: OR: Odds ratio; HR: harzard ratio; CD: *Clostridium difficile*; LOS: length of stay; ICD-9: 9^th^ International classification of diseases; PPI: proton pump inhibitor; H2RA: histamine receptor antagonist; VA: Veteran Affairs; D: Durham County, TPN: Total parenteral nutrition
