# Supplementary material for: Association between Proton Pump Inhibitor Therapy and Clostridium difficile Infection: A Contemporary Systematic Review and Meta-Analysis
Source: PLoS One. 2012 Dec 7;7(12):e50836. doi: 10.1371/journal.pone.0050836 (PMC3517572; doi:10.1371/journal.pone.0050836)
Supplement: Table S2 — The Association between PPI use and Development of Clostridium difficile infection from Cohort citations. (DOCX) [file pone.0050836.s002.docx]

| **Table S2. The Association between PPI use and Development of *Clostridium difficile* infection from Cohort citations** | | | | |
| --- | --- | --- | --- | --- |
| **Adjusted Effect Estimates** | **Sample Size** | **Selection of Controls** | **Case Ascertainment** | **Source** |
| HR : 1.42 (1.10-1.83) | Exposed group; cases: 133, controls: 394  Non-exposed group; cases:118, controls: 521 | CDI with no PPI exposure concurrent with CDI treatment | Diarrhea:Stool positive for CD toxin | Linsky et al,^35^ 2010 |
| Daily PPI  OR: 1.74 (1.39 -2.18); PPI more than daily  OR: 2.36 (1.79-3.11) | Daily PPI  Exposed group; cases: 360, controls: 40250  Non-exposed group; cases:305,controls: 60881  PPI more than daily  Exposed group; cases: 134, controls: 9102  Non-exposed group; cases:531,controls: 92029 | A nearest-neighbor–matching algorithm was applied. | Positive CD toxin | Howell et al,^36^ 2010 |
| PPI days  OR : 1.01 (1.00-1.02) | Exposed group; cases: 83, controls: 5688  Non-exposed group; cases:66, controls: 8882 | Age, ≥ 18 years, Minimum 7d LOS, Antibiotic exposure | Positive CD toxin, colonoscopy proven pseudomembranous colitis | Dalton et al,^37^ 2009 |
| OR :1.6 (1.3-2.1) | Exposed group; cases: 267, controls: 13476  Non-exposed gp; cases: 115, controls: 22228 | In-patient, No positive stool toxin assay during the period (60 day before start of study to the end) | Positive stool for CD toxin | Dubberk et al,^38^ 2007 |
| HR, 1.0 (0.79-1.28) | Exposed group; cases: 3134, controls: NR  Non-exposed group; cases: 4287, controls: NR | Unclear | Diarrhea, Positive toxin, proven pseudomembranous colitis | Pépin et al,^39^ 2005 |
| HR, 0.90 (0.59-1.38) | Exposed group; cases: 335, controls: NR  Non-exposed group; cases: 492, controls: NR | Unclear | Diarrhea  Positive stool for CD toxin | Beaulieu et al,^40^ 2007 |
| OR, 6.1 p Value 0.024 | Exposed group; cases: 11, controls: 11  Non-exposed group; cases: 41, controls: 154 | Diarrhea with negative stool for CD, same institution | Diarrhea  Positive stool for CD toxin | Peled et al,^41^ 2007 |
| OR, 2.1 (1.2-3.5) | Exposed group; cases: 55, controls: 536  Non-exposed group; cases: 26, controls: 570 | Unclear | Positive stool for CD toxins | Dial et al,^17^ 2004 |
| Crude OR= 1.26, P value = 0.33 | Exposed group; Cases =11, Control=57  Non-exposed group; Cases: 14, Control: 92 | All patients with CDI in the same institution | Diarrhea between 5-60 days after the initial antibiotic course for CDI | Netland et al,^42^  2011 |
| OR = 1.84, CI (0.45-7.45), p value 0.38 | Exposed group; Case=7, Control=16  Non-exposed group; Cases: 10, Control: 66 | Diarrhea with negative stool for CD toxins, Same institution | Diarrhea, Positive CD toxin | Ingle et al,^43^ 2011 |
| HR=1.11, CI (0.44-2.78), P Value=0.83 | Exposed group; NR  Non-exposed group; NR | Matched to the unit and study period | Positive CD toxins | Shaughnessy et al,^44^ 2011 |
| OR = 3.48, CI (1.64-7.69), P value = 0.016 | Exposed group; Case=17, Control=39  Non-exposed group; Cases: 10, Control: 59 | Same institution | Diarrhea, Positive CD toxin | Kim et al,^45^ 2010 |
| OR=2.16, CI(1.03-4.56) | Exposed group; NR  Non-exposed group; NR | Frequency matching approach | Diarrhea and: Positive CD toxin/culture or pseduomembranous colitis or histological diagnosis | Loo et al,^46^ 2011 |
| HR=4.5, CI(2.3-9), P Value= < 0.000 | Exposed group; Cases=201, Control=6121  Non-exposed group; Cases: 40, Control: 3832 | Same institution | Diarrhea, Positive CD toxin | Stevens et al,^47^ 2011 |

NB: OR: Odds ratio; HR: Harzard ratio; CD: *Clostridium difficile*; LOS: PPI: Proton pump inhibitor; NR: Not reported
