# Supplementary material for: Association between Proton Pump Inhibitor Therapy and Clostridium difficile Infection: A Contemporary Systematic Review and Meta-Analysis
Source: PLoS One. 2012 Dec 7;7(12):e50836. doi: 10.1371/journal.pone.0050836 (PMC3517572; doi:10.1371/journal.pone.0050836)
Supplement: Table S3 — Meta-regression analysis to explore sources of heterogeneity. (DOCX) [file pone.0050836.s003.docx]

| **Table S3.** Meta-regression analysis to explore sources of heterogeneity | | | | |
| --- | --- | --- | --- | --- |
|  | **Univariate Analyses** | | **Multivariate Analyses** | |
| **Study Characteristics** | **Coefficient** | **p-values** | **Coefficient** | **p-values** |
| Study Design | -0.0061 | 0.97 |  |  |
| Country where the study is conducted |  |  |  |  |
| *European countries* | Reference |  |  |  |
| *Canada* | -0.1663 | 0.341 |  |  |
| *United States* | 0.1399 | 0.506 |  |  |
| *Asian Countries* | 0.6247 | 0.121 |  |  |
| Setting | -0.00239 | 0.99 |  |  |
| No of variables adjusted for | 0.001527 | 0.927 |  |  |
| Impact factor of the journal | 0.00099 | 0.89 |  |  |
| Method of ascertainment of antibiotic |  |  |  |  |
| *Patient chart* | Reference |  |  |  |
| *Pharmacy record* | -0.24978 | 0.241 |  |  |
| *Interview* | -0.4501 | 0.034 | -0.4059 | 0.051 |
| *Questionnaire* | 0.69996 | 0.273 |  |  |
| *Combined* | 0.1013 | 0.656 |  |  |
| *Not reported* | -0.0256 | 0.94 |  |  |
| Proportion of antibiotic use | 0.00117 | 0.750 |  |  |
| Effect estimate measure (OR vs. other) | 0.2111 | 0.345 |  |  |
| Adjusted vs. unadjusted estimates | -0.3637 | 0.047 | -0.325665 | 0.079 |
|  |  |  |  |  |
