# Supplementary material for: Association between Proton Pump Inhibitor Therapy and Clostridium difficile Infection: A Contemporary Systematic Review and Meta-Analysis
Source: PLoS One. 2012 Dec 7;7(12):e50836. doi: 10.1371/journal.pone.0050836 (PMC3517572; doi:10.1371/journal.pone.0050836)
Supplement: Table S4 — Forest plot of the meta-analysis of the proportion of Clostridium difficile cases that were exposed to antibiotics. (DOCX) [file pone.0050836.s004.docx]

| **Table S4. Modified Newcastle-Ottawa Quality Assessment Scale for Cohort studies included in the Meta-analysis** | | | | | | | | |
| --- | --- | --- | --- | --- | --- | --- | --- | --- |
| **Included Studies** | **Selection*** | | | | **Comparability^•^** | **Outcome⁰** | | |
|  | **Representativeness of the exposed cohort** | **Selection of the Non-exposed Cohort** | **Ascertainment of Exposure** | **Incident**  **Disease** |  | **Assessment**  **of**  **Outcome** | **Length of**  **Follow-up** | **Adequacy of Follow-up** |
| Linsky et al,^35^ 2010 | A | A | A | A | A | A | A | A |
| Howell et al,^36^ 2010 | A | A | A | A | A | B | A | A |
| Dalton et al,^37^ 2009 | A | A | A | A | A | B | A | A |
| Dubberke et al,^38^ 2007 | A | A | A | A | A | B | A | A |
| Pépin et al,^39^ 2005 | A | A | A | A | A | B | A | A |
| Beaulieu et al,^40^ 2007 | B | A | A | A | A | A | A | A |
| Peled et al,^41^ 2007 | A | A | B | A | A | A | A | A |
| Dial et al,^17^ 2004 | A | A | A | A | A | A | A | A |
| Netland et al,^42^ 2011  Ingle et al,^43^ 2011  Kim et al,^45^ 2010  Stevens et al,^47^ 2011  Shaughnessy et al,^44^ 2011 | A  A  A  A  A | A  A  A  A  A | A  A  A  A  A | A  A  A  A  A | A  A  A  A  A | B  B  B  B  B | A  A  A  A  A | A  A  A  A  A |

*Abbreviations: NR, Not Reported.*

**Selection:*

*(1) Representativeness of the exposed cohort: A, truly representative; B, somewhat representative; C, selected group; D, no description of the derivation of the cohort*

*(2) Selection of the non-exposed cohort: A, drawn from the same community as the exposed cohort; B, drawn from a different source; C, no description of the derivation of the non exposed cohort.*

*(3) Ascertainment of exposure: A, secure record; B, structured interview; C, written self-report; D, no description.*

*(4) For demonstration that the outcome of interest was not present at start of study: A, yes; B, no.*

*^•^Comparability: For comparability of cohorts on the basis of the design or analysis: A, study controls for co-morbidities; B, study controls for any additional factor (e.g., age and severity of illness); C, not done.*

*⁰Outcome:*

*(1) Assessment of outcome: A, independent blind assessment; B, record linkage; C, self-report; D, no description.*

*(2) Was follow-up long enough for outcomes to occur? A, yes (i.e. in-hospital or up to 30 d); B, no.*

*(3) Adequacy of follow-up of cohorts: A, complete follow-up and all subjects accounted for; B, subjects lost to follow-up was unlikely to introduce bias, because a small number were lost or a description was provided of those lost; C, follow-up rate 90% or lower (select an adequate percentage) and no description of those lost; D, no statement.*
