# Supplementary material for: Association between Proton Pump Inhibitor Therapy and Clostridium difficile Infection: A Contemporary Systematic Review and Meta-Analysis
Source: PLoS One. 2012 Dec 7;7(12):e50836. doi: 10.1371/journal.pone.0050836 (PMC3517572; doi:10.1371/journal.pone.0050836)
Supplement: Table S5 — Modified Newcastle-Ottawa Quality Assessment Scale for Case-control studies included in the Meta-analysis. (DOCX) [file pone.0050836.s005.docx]

| **Table S5. Modified Newcastle-Ottawa Quality Assessment Scale for Case-control studies included in the Meta-analysis** | | | | | | | | |
| --- | --- | --- | --- | --- | --- | --- | --- | --- |
| **Included Studies** | **Selection*** | | | | **Comparability^•^** | **Exposure⁰** | | |
|  | **Adequacy of Case Definition** | **Representativeness of the Cases** | **Selection of Controls** | **Definition**  **of**  **Controls** |  | **Ascertainment**  **of**  **Exposure** | **Same Method of**  **Ascertainment for**  **Cases and Controls** | **Non-Response**  **Rate** |
| Kutty et al,^1^ 2010 | A | A | A | A | A | A | A | NR |
| Southern et al,^2^ 2010 | A | A | B | B | A | A | A | NR |
| Nath et al,^3^ 1994 | A | A | B | A | A | A | A | NR |
| Jayatilaka et al,^4^ 2007 | B | A | B | A | A | A | A | NR |
| Branch et al,^5^ 2007 | A | A | B | A | A | A | A | NR |
| Al-Tureihi et al,^6^ 2005 | A | A | B | A | A | A | A | NR |
| Baxter et al,^7^ 2008 | A | A | B | A | A | A | A | NR |
| Shah et al,^8^ 2000 | A | A | B | A | A | A | A | NR |
| Lowe et al**,**^9^ 2006 | A | A | A | A | A | A | A | NR |
| Yearsley et al,^10^ 2006 | A | A | B | A | A | A | A | NR |
| Dial et al,^11^ 2005 | A | A | A | A | A | A | A | NR |
| Akhtar et al,^12^ 2007 | A | A | B | A | A | A | A | NR |
| Dial et al,^13^ 2006 | B | A | A | A | A | A | A | NR |
| Aseeri et al,^14^ 2008 | A | A | B | A | A | E | B | NR |
| Cunningham et al,^15^ 2002 | A | A | B | B | A | A | A | NR |
| Dubberke et al,^16^ 2007 | A | B | B | A | A | A | A | NR |
| Dial et al,^17^ 2004 | A | A | B | B | A | A | A | NR |
| Loo et al,^18^ 2005 | A | A | B | A | A | E | A | NR |
| Sundram et al,^19^ 2009 | A | A | B | A | A | A | A | NR |
| Debast et al,^20^ 2009  Novell and Moreale,^21^ 2010  Hensgens et al,^22^ 2011  Jenkins et al,^23^ 2010  Kuntz et al,^26^ 2011  Loo et al,^46^ 2011  Manges et al,^24^ 2010  Naggie et al,^25^ 2011  Monge et al,^27^ 2011   \| Linney et al,^39^ 2010  McFarland et al,^29^ 2007  Modena et al,^31^ 2005 \| \| --- \| \| Muto et al,^32^ 2005 \| \| Yip, et al,^33^ 2001 \| \| Dial et al,^28^ 2008  Kazakova et al,^30^ 2006 \| | A  A  A  B  A  A  A  A  A  B  B  B  B  B  B  B | A  A  A  A  A  A  A  A  A  A  A  A  A  A  A  A | B  B  B  B  A  B  B  A  B  B  C  B  B  B  B  B | A  A  A  A  A  A  B  A  A  B  A  A  A  A  A  A | A  A  A  A  A  A  A  A  A  A  A  A  A  A  A  A | A  A  A  A  A  A  A  C  A  A  A  A  A  A  A  A | A  A  A  A  A  A  A  A  A  A  A  A  A  A  A  A | NR  NR  NR  NR  NR  NR  NR  NR  NR  NR  NR  NR  NR  NR  NR  NR |

*Abbreviations: NR, Not Reported.*

**Selection:*

*(1)Is this case definition adequate? A, yes, with independent validation; B, yes, eg record linkage or based on self reports C, no description*

*(2) Representativeness of the cases: A, Consecutive or obviously representative series of cases; B, Potential for selection biases or not stated*

*(3) Selection of controls: A, Community controls; B, Hospital controls; C, No description*

*(4) Definition of controls: A, No history of disease; B,No description of source*

*^•^Comparability: Comparability of cases and controls on the basis of the design or analysis: A, study controls for co-morbidities; B, study controls for any additional factor (e.g., age and severity of illness)*

*⁰Exposure:*

*(1) Ascertainment of exposure: A, Secured records; B,Structured interview where blind to case/control status; C, Interview not blinded to case/control status; D,written self report or medical record only*

*(2) Same method of ascertainment for cases and controls; A, yes; B, no.*

*(3) Non-response rate: A,Same for both groups; B, Non-respondents described; C, Rate different and no designation*
