# Supplementary material for: Association between Proton Pump Inhibitor Therapy and Clostridium difficile Infection: A Contemporary Systematic Review and Meta-Analysis
Source: PLoS One. 2012 Dec 7;7(12):e50836. doi: 10.1371/journal.pone.0050836 (PMC3517572; doi:10.1371/journal.pone.0050836)

**Figure S1.** Forest plot of the meta-analysis of the proportion of *Clostridium difficile* cases that were exposed to antibiotics


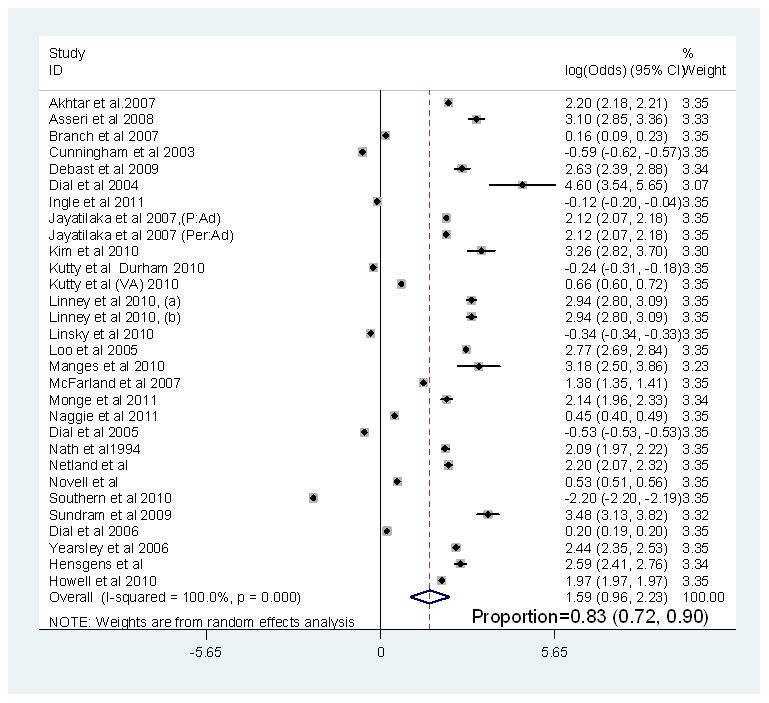

Supplement: Figure S1 — Forest plot of the meta-analysis of the proportion of Clostridium difficile cases that were exposed to antibiotics. (DOCX) [file pone.0050836.s006.docx]
